# Sleeping beauty generated CD19 CAR T-Cell therapy for advanced B-Cell hematological malignancies

**DOI:** 10.3389/fimmu.2022.1032397

**Published:** 2022-11-10

**Authors:** Harjeet Singh, Samer A. Srour, Denái R. Milton, Jessica McCarty, Cuiping Dai, Mahmoud R. Gaballa, Mariam Ammari, Simon Olivares, Helen Huls, Eleanor De Groot, David Marin, Demetrios Petropoulos, Amanda L. Olson, Paolo Anderlini, Jin S. Im, Issa Khouri, Chitra M. Hosing, Katayoun Rezvani, Richard E. Champlin, Elizabeth J. Shpall, Laurence J. N. Cooper, Partow Kebriaei

**Affiliations:** ^1^ Department of Pediatrics, The University of Texas MD Anderson Cancer Center, Houston, TX, United States; ^2^ Department of Stem Cell Transplantation and Cellular Therapy, The University of Texas MD Anderson Cancer Center, Houston, TX, United States; ^3^ Department of Biostatistics, The University of Texas MD Anderson Cancer Center, Houston, TX, United States; ^4^ Cellular Therapy Program and Bone Marrow Transplant Unit, Massachusetts General Hospital Cancer Center, Harvard Medical School, Boston, MA, United States; ^5^ Alaunos Therapeutics, Boston, MA, United States

**Keywords:** sleeping beauty, non-viral gene transfer, CD19, CAR, T cells, lymphoid malignancy, acute lymphoblastic leukemia, non-hodgkin lymphoma

## Abstract

Chimeric antigen receptor (CAR) T-cell therapy has emerged recently as a standard of care treatment for patients with relapsed or refractory acute lymphoblastic leukemia (ALL) and several subtypes of B-cell non-Hodgkin lymphoma (NHL). However, its use remains limited to highly specialized centers, given the complexity of its administration and its associated toxicities. We previously reported our experience in using a novel Sleeping Beauty (SB) CD19-specific CAR T-cell therapy in the peri-transplant setting, where it exhibited an excellent safety profile with encouraging survival outcomes. We have since modified the SB CD19 CAR construct to improve its efficacy and shorten its manufacturing time. We report here the phase 1 clinical trial safety results. Fourteen heavily treated patients with relapsed/refractory ALL and NHL were infused. Overall, no serious adverse events were directly attributed to the study treatment. Three patients developed grades 1-2 cytokine release syndrome and none of the study patients experienced neurotoxicity. All dose levels were well tolerated and no dose-limiting toxicities were reported. For efficacy, 3 of 8 (38%) patients with ALL achieved CR/CRi (complete remission with incomplete count recovery) and 1 (13%) patient had sustained molecular disease positivity. Of the 4 patients with DLBCL, 2 (50%) achieved CR. The SB-based CAR constructs allow manufacturing of targeted CAR T-cell therapies that are safe, cost-effective and with encouraging antitumor activity.

## Introduction

Despite the advances made over the past decade and the introduction of several novel therapeutics, there remains an unmet need to further improve the outcomes of patients with advanced hematologic malignancies. CD19-targeted chimeric antigen receptor (CAR) T-cell therapy has emerged recently as one of the new standard treatments for patients with relapsed or refractory acute lymphoblastic leukemia (ALL) and several subtypes of B-cell non-Hodgkin lymphoma (NHL) (1–4). However, durable responses are noted in less than 50% of these patients (5), and the widespread use of this promising therapy is hampered by the known unique and potentially serious toxicities, particularly cytokine release syndrome (CRS) and immune effector cell-associated neurotoxicity syndrome (ICANS) (6). Hence, there is a need for CAR constructs with a better safety profile that at a minimum maintain this practice-changing therapy’s efficacy, if not improve it.

We have previously reported our experience in using a novel Sleeping Beauty (SB) (7) CD19-specific CAR T-cell therapy in two phase 1 clinical trials (NCT00968760 and NCT01497184) (8, 9). T-cells were genetically modified using the SB transposon/transposase system to produce a second-generation CAR construct (10), with co-signaling through CD3 and CD28 (11). Through incorporating SB CAR T-cells into the hematopoietic stem cell transplantation (HCT) setting (infusing cells 2 days after stem cell infusion), we showed an excellent safety profile, long-term persistence of the genetically modified T-cells (median of 4.5 years), and potentially improved outcomes in patients with advanced B-cell lymphoid malignancies (8, 12). Twenty-six patients were treated in these phase 1 studies, with no unexpected acute or delayed toxicities noted. Based on the promising phase 1 findings in patients with low tumor burden at time of cell infusion, we made modifications to the CAR stalk to reduce binding to Fc receptors, and modifications to the manufacturing process to shorten the production time in efforts to improve efficacy in patients with bulky disease, and improve ease of administration, respectively (13). Herein, we report the final safety and efficacy results of this clinical trial (NCT02807883; IND# 16474).

## Materials and methods

### Study design

This was a prospective, open-label, single-arm, single center, phase 1 clinical trial evaluating 5 dose escalation/de-escalation levels (DL -1: ≤ 1 x 10^5^/kg; DL +1 > 1 x 10^5^/kg but ≤ 1 x 10^6^/kg; DL +2 > 1 x 10^6^/kg but ≤ 1 x 10^7^/kg; DL +3 > 1 x 10^7^/kg but ≤ 1 x 10^8^/kg; DL +4 > 1 x 10^8^/kg but ≤ 1 x 10^9^/kg). The phase 1 clinical trial design we employed in this study was as previously described by Ji et al. (14). Dose-limiting toxicity (DLT) was defined as a non-reversible grade 3 or any grade 4-5 non-hematological organ toxicities and/or allergic/autoimmune reactions related to the study cell infusion. Adverse events were graded using the Common Terminology Criteria for Adverse Events version 4 (CTCAE V 4.0). Responses were assessed and defined per disease category, as previously described (15).

### Ethics approval and patient consent

The study was conducted after the protocol was reviewed and approved by MD Anderson Cancer Center’s Institutional Review Board (IRB). Patients provided informed consent prior to enrollment in the clinical study in accordance with the Declaration of Helsinki. This phase 1 clinical trial was registered at ClinicalTrials.gov (NCT02807883).

### Patient eligibility

Patients with relapsed/refractory CD19^+^ B-cell lymphoid malignancies, ages 1 through 80 years, were eligible. B-cell lymphoid malignances included acute lymphoblastic leukemia (ALL), diffuse large B-cell lymphoma (DLBCL), chronic lymphocytic leukemia/small lymphocytic lymphoma (CLL/SLL), follicular lymphoma, marginal zone lymphoma, and mantle cell lymphoma, with confirmed positive CD19 by flow cytometry on the malignant cells. At study entry, patients were required to have adequate organ function, Karnofsky performance status (KPS) >60%, and with no evidence of active hepatitis B or C infection. Patients with a history of HIV infection were excluded. Those with prior allogeneic HCT were allowed after at least 3 months following transplant. Patients had measurable disease at study entry and had failed standard frontline therapy. Bridging chemotherapy to control disease while waiting for CAR T-cell production was allowed and at the discretion of the treating physician. Notably, patients must have had measurable disease and adequate organ function at time of starting lymphodepletion prior to CAR T infusion.

### DNA constructs

This study used a second generation CD19-specific CAR (10, 16, 17) consisting of an anti-CD19scfv held on the cell surface by a CD8α stalk and with signaling through CD3ζ and CD28 co-stimulatory endodomains (CD19RCD8CD28) (13). The CAR was expressed in a SB transposon and SB transposase was encoded by a pCMV-SB11 plasmid (18).

### Cell lines

All cell lines were cultured in complete media (RPMI 1640, 10% heat inactivated fetal bovine serum (HyClone), and 1% Glutamax-100 (Gibco)) at 5% CO_2_ and 37°C. Daudiβ_2_m, NALM-6, EL-4 and EL-4 modified to express CD19 (CD19^+^ EL-4) were maintained, as previously described (18). K562 clone #1 AaPC was developed as previously described (19) and expressed CD19, CD32, CD64, CD86, CD137L and membrane bound IL-15. A working cell bank of clone #1 was used to propagate AaPC in WAVE Bioreactors, γ-irradiated, and cryopreserved for future use, as described previously (8). Thawed irradiated AaPC were utilized for generation of CAR T-cells. Cell lines were negative for mycoplasma and endotoxin. The identities of cell lines were established by STR DNA fingerprinting performed by the Characterized Cell Line Core at MD Anderson Cancer Center.

### Generation of CAR T-cells

Peripheral blood mononuclear cells (PBMC) were obtained *via* patient derived leukapheresis products. PBMC were isolated using the Biosafe Sepax II platform, as described previously (18). In short, the Sepax II is a closed system centrifugation instrument that utilizes an automated Ficoll gradient protocol to separate PBMCs from non-target cell types. After Ficoll gradient isolation, PBMCs were washed twice with PBS/EDTA supplemented with human serum albumin and the washed cells were transferred into a 200 mL blood banking bag. The PBMCs were then cryopreserved for future manufacturing purposes. Genetic modification to generate CAR T-cells was performed, as described earlier (8). PBMC were thawed and rested in complete media for two hours at 37°C, 5% CO_2_. The rested cells were resuspended at a concentration of 2x10^7^/100µL of a mixture containing 15µg transposon DNA plasmid coding for CD19RCD8CD28 transposon, 5µg transposase DNA plasmid (pCMV-SB11) coding for SB11 transposase, and Human T-cell kit reagent (cat# VPA-1002, Lonza). The mixture was transferred to a cuvette, electroporated using program U-14 of the Nucleofector II device (Amaxa, Lonza) and transferred to complete media for a two-hour rest at 37°C, 5% CO_2_. A half media change was performed and the electroporated cells were then incubated overnight at 37°C, 5% CO_2_. The next day, cells were harvested, counted, and phenotyped by flow cytometry. Cells were then co-cultured with 100Gy irradiated K562 clone #1 AaPCs at a 1:1 ratio (AaPC: CAR^+^ T-cell) along with IL-21 (PeproTech, 30ng/mL). Media changes and cytokine additions were performed every 2-3 days. IL-2 (Aldesleukin, Novartis, 50U/mL) was incorporated into the media changes starting at day 7 to avoid early outgrowth of natural killer (NK) cells. T-cell cultures were evaluated for CAR^+^ expression and re-stimulated every 7 days with 100Gy irradiated clone #1 AaPCs with the addition of IL-21 and IL-2. T-cells were expanded in culture to reach appropriate patient dose levels and cryopreserved thereafter.

### Lymphodepletion and CAR T-cell infusion

Lymphodepletion was recommended for all study patients, unless there were remarkable cytopenias from prior therapies. Lymphodepletion consisted of fludarabine 30 mg/m^2^ and cyclophosphamide 500 mg/m^2^ for 3 consecutive days, followed by CAR T-cell infusion at least 48 hours after completion of lymphodepletion. Reduced intensity lymphodepletion (fludarabine 25 mg/m^2^ and cyclophosphamide 250 mg/m^2^) was allowed at the discretion of the treating physician. Prior to CAR T-cell infusion, patients should have been off steroids for at least 72 hours (unless on physiological dose replacements), with no active infection, and with resolution of any non-hematologic toxicity from lymphodepletion to < grade 3. The day of CAR T-cell infusion was designated as Day 0. The CAR T-cell dose was defined by the dose group per the phase 1 dose escalation/de-escalation schedule, as described in the study design section.

### Safety and evaluations

Disease assessments with peripheral blood studies, bone marrow examinations, and PET when clinically relevant were done prior to study entry and at 30 days following CAR T infusion to assess for response. The CTCAE V 4.0 was used to grade toxicities.

### Response definitions and outcome measures

The primary objectives were to determine the safety profile and maximum tolerated dose (MTD) of SB CAR T-cells. Secondary objectives included assessment of disease response and to determine persistence of CAR T-cells. CR was defined as having ≤ 5% malignant blasts in the bone marrow, recovery of normal blood counts with absolute neutrophil count ≥ 0.5 x 10^9^/L and platelet count > 20 x 10^9^/L, normal karyotype, and absence of extramedullary disease. MRD was assessed using multiparameter flow cytometry with a threshold of > 0.01%. CR for lymphoma was defined by CT and/or PET, as per Cheson criteria (15).

### Flow cytometry

Immunophenotyping by flow cytometric analysis was performed by staining T-cell suspensions with a live/dead stain followed by surface antibody staining for anti-CD3, CD4, CD8, CD45, CD56, CD11c, CD19, CD14, CD16, CD20, CD32, CD45RO, CD27, CD95, CD45RA, CD28 CD62L, CD197, TCRαβ, TCRγδ and CAR (**Supplementary Methods**). All experiments were performed using a BD Fortessa or BD FACS Calibur. Data was analyzed using FlowJo software.

### Chromium release assay

Specific lysis of CD19^+^ targets by CAR^+^ T-cells was determined using a standard 4 hour chromium release assay (18).

### Gene integration (CAR copy number)

Assessment of integrated CAR copy number of SB-modified T-cells was determined by droplet digital PCR (ddPCR), a sensitive method of detecting and quantifying infrequent target DNA molecules (20), as described previously (8). 50ng of genomic DNA was multiplexed using primer/probe sets for the CAR and a housekeeping gene (EIF2C1) (**Supplementary Methods**). PCR droplets were generated and analyzed using a QX-100 Digital Droplet PCR System (Bio-Rad).

### Serum cytokines

Blood samples collected post CAR T-cell infusion were processed to isolate serum and cryopreserved in aliquots at -80°C for analysis. For evaluation of cytokines in the serum of patients post infusion, serum samples were thawed and processed using a Bio-Plex Pro Human Cytokine 27-plex Assay (Bio-Rad, Hercules, CA) according to the manufacturer’s instructions. Briefly, serum samples were diluted (1:4) in complete media (RPMI containing 10% FBS with Glutamax-1), incubated with capture beads, and read in a Bio-Plex 200 system (Bio-Rad).

### Statistical analysis

Progression-free survival (PFS) time was computed from date of cell infusion to date of progression or death, whichever came first. Patients who were alive at their last follow-up date who had not progressed were censored. Overall survival (OS) time was computed from date of cell infusion to date of death. Patients who were alive at their last follow-up date were censored. The Kaplan-Meier method was used to estimate PFS and OS.

## Results

### Generation of CAR T-cells for clinical trial

**Figure 1:**
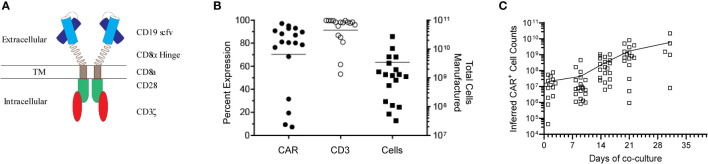
Design and characterization of the CD19 CAR. **(A)** Schematic of the CD19-specific CAR, which was held on the cell surface *via* a CD8α hinge stalk and signaled through CD28 and CD3ζ endodomains. **(B)** Characterization of the infusion product. Patient derived CAR^+^ T-cells were generated by co-culture of genetically modified T-cells with K562 AaPCs. Cells were phenotyped and enumerated every 7 days. The total cells generated and percent CAR and CD3 expression of the cell products at time of cryopreservation is shown. **(C)** Expansion kinetics of all the manufactured patient derived CAR^+^ T-cells over time. Each symbol represents an individual patient.

Genetically modified T-cells were co-cultured with γ-irradiated AaPCs (clone #1) in the presence of exogenous cytokines (IL-2, IL-21) for a median of 22 days (mean ± SD, 23.4 ± 5.19) and cryopreserved for infusion (**Figure 1A**). Twenty-six patients were enrolled on the study from June 2016 through April 2019. CAR product was successfully generated for 23 patients, while product manufacture failure occurred for 3 patients (**Supplementary Table S1**). The gene-modified T-cells had an average expansion of 4681-fold for CAR^+^ T-cells, median 97.9% CD3^+^ (mean ± SD, 91.4% ± 13.26%), median 80.5% CAR^+^ (mean ± SD, 70.1% ± 29.7%) and were predominantly CD8^+^ (mean ± SD, 70.2% ± 18.7%; mean CD4/CD8, 0.19) (**Figures 1B, C**, **Supplementary Table S1**). They were able to effectively lyse at a effector:target ratio of 5:1 various CD19^+^ B-cell lines, Daudi (Burkitt’s Lymphoma) co-expressing β_2_-microglobulin (21) (Daudiβ_2_m) to reduce lysis by LAC or NK cells (mean ± SD, 44.6% ± 18.2%) and NALM-6 (pre-B ALL; mean ± SD, 38.4% ± 15.1%) in a cytotoxicity assay. An increase of 4.8-fold (5:1, E:T) in killing of CD19^+^ EL-4 cells as compared to unmodified CD19^neg^ EL-4 (mouse T-cell lymphoma) targets demonstrated specificity for CD19 by CAR T cells. (**Supplementary Figure S1, Supplementary Table S2**). Expanded T cells were cryopreserved, passed release testing to generate a certificate of analysis (22), and were thawed on the day of infusion after the recipient met eligibility.

### Patient characteristics and safety

Fourteen patients with a median age of 40 years (range, 16-73 years) received CAR T-cell therapy and were included in the final safety and efficacy analysis (**Table 1**). Nine patients did not have cells infused due to the following reasons: rapid disease progression with clinical deterioration and death (n=4), no disease at time of CAR availability (n=3, 2 of which remain in remission and 1 patient relapsed with CNS involvement and died from disease progression), allogeneic HCT (n=1), and loss of insurance (n=1).

**Table 1:** Study patient characteristics and treatment outcomes in detail for the infused patients, N=14.

Acc #	Age	Gender	Diagnosis	Prior lines of therapy	Prior transplant	Cohort	CRS	ICANS	Response	Progressed	Status at last follow-up
**2**	68	M	CLL	3	No	1	No	No	No response	Yes	Died, secondary cancer
**4**	40	M	ALL	6	Yes	1	No	No	CRi	Yes	Died, active disease
**6**	36	F	DLBCL	4	Yes	-1	No	No	No response	Yes	Died, active disease
**8**	40	F	ALL	4	Yes	1	No	No	CR	Yes	Died, active disease
**9**	29	F	ALL	4	No	2	Yes	No	No response	Yes	Died, active disease
**13**	46	M	ALL	5	Yes	2	Yes	No	MRD negative	Yes	Died, active disease
**14**	72	M	DLBCL	5	Yes	2	Yes	No	CR	No	Alive, in remission
**16**	16	M	ALL	3	Yes	1	No	No	No response	Yes	Died, active disease
**20**	47	F	DLBCL*	3	No	3	No	No	CR	Yes	Alive, in remission
**21**	31	M	ALL	4	Yes	3	No	No	No response	Yes	Died, active disease
**22**	73	F	DLBCL	2	No	-1	No	No	Progression	Yes	Died, active disease
**23**	57	F	CLL	3	Yes	3	No	No	No response	Yes	Alive, active disease
**25**	34	M	ALL	4	Yes	1	No	No	No response	Yes	Died, active disease
**26**	39	M	ALL	2	Yes	1	No	No	MRD positive	No	Alive, in remission

ALL, acute lymphoblastic leukemia; CLL, chronic lymphocytic leukemia; CR, complete remission; CRi, CR with incomplete platelet and/or neutrophil recovery; CRS, cytokine release syndrome; ICANS, immune effector cell associated neurotoxicity syndrome; DLBCL, diffuse large B-cell lymphoma; F, female; M, male; MRD, minimal residual disease.

*Transformed from marginal zone lymphoma.

Of the 14 patients who received thawed SB CD19-specific CAR T-cells (viability, mean ± SD, 98.8% ± 2.13%, **Supplementary Table S1**), 8 patients had B-ALL, 4 patients had DLBCL and 2 patients had CLL. Patients were heavily pretreated, with a median of 4 (range, 2-6) prior lines of therapy. Additionally, 10 patients had prior allogeneic HCT, of whom 3 received two transplants (one patient received an autologous and then an allogeneic transplant, and two patients received two prior allogeneic transplants). Two patients, numbers 2 and 16 in **Table 1**, received reduced intensity lymphodepletion due to significant cytopenia at time of study treatment.

Overall, no serious adverse events were directly attributed to the study treatment. Only 1 patient had a grade 2 infusion reaction, which resolved with supportive treatment. No unexpected acute or delayed toxicities were observed. Three patients developed grades 1-2 CRS and none of the study patients had ICANS. Three events of grade 3 non-hematological adverse events occurred; one each for infection, elevated alanine aminotransferase, and elevated aspartate aminotransferase. All dose levels were well tolerated with no DLTs reported.

### Response and survival

**Figure 2:**
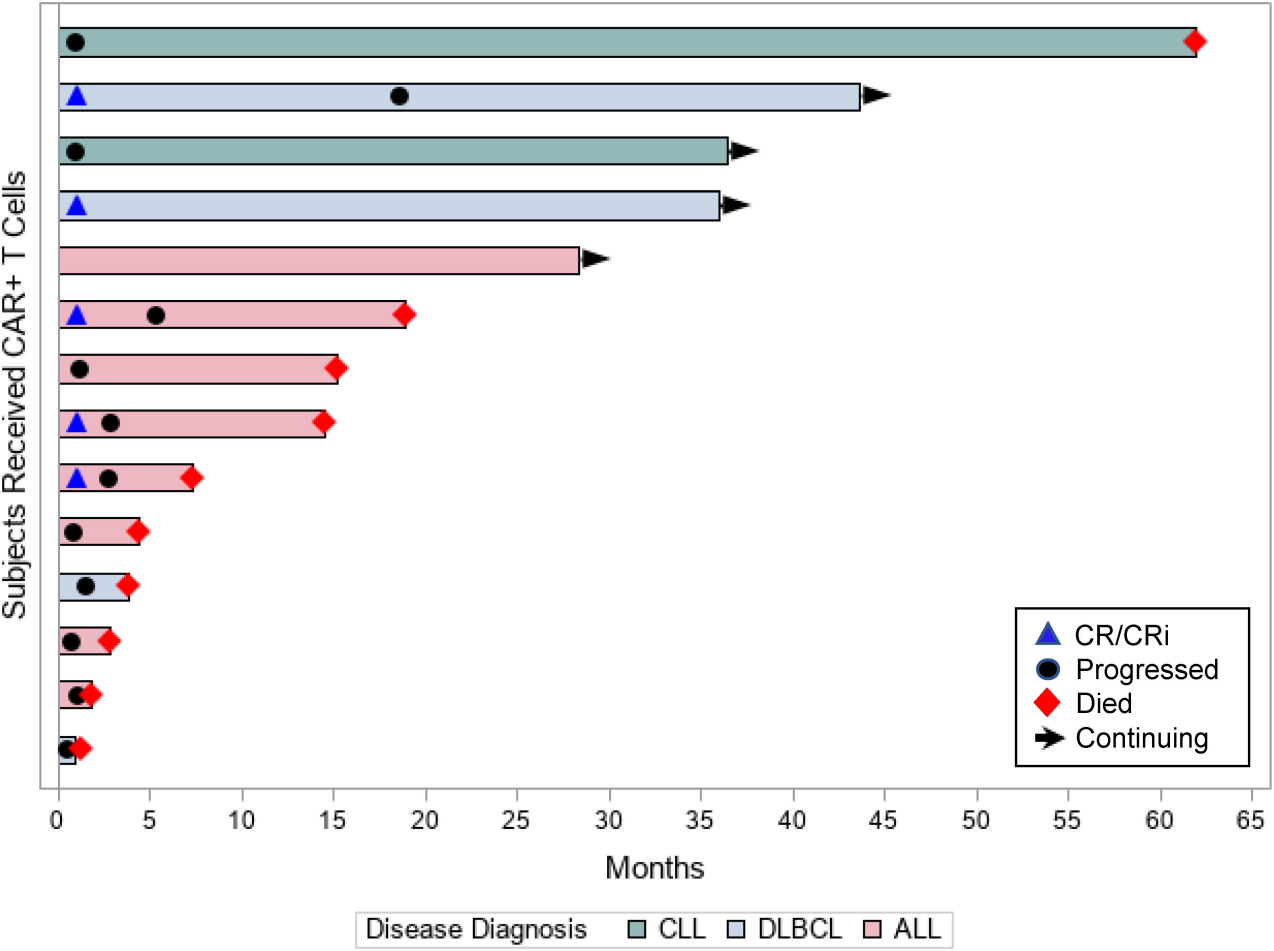
Patient responses. Swimmer’s plot displays each individual patient’s time in the study (in months), their disease diagnosis, and outcomes.

Of the 14 patients assessed for efficacy, 5 (36%) patients achieved objective responses, 2 (14%) had stable disease (both had CLL), and 1 patient had sustained minimal residual disease (MRD) positivity (ALL patient). **Table 1** presents the disease and treatment characteristics of the 14 individual patients in the study and their respective outcomes. Of the 8 patients with ALL, 3 (38%) patients achieved CR/CRi (complete remission with incomplete count recovery) at 1 month, 1 (13%) patient had sustained molecular measurable residual disease (MRD) positivity, and 4 (50%) patients had no response. Of the responding 3 patients in remission, all progressed during the study period (**Figure 2**). Of the 4 patients with DLBCL, 2 (50%) achieved complete remission, 1 patient had progressive disease at 1 month after CAR T-cell therapy, and 1 patient had rapid leptomeningeal central nervous system progression and transitioned to hospice before day 30 disease assessments. The 2 responding patients had durable remissions; 1 remains in remission at 3 years after cell infusion and the second patient progressed at 18 months after therapy (**Figure 2**). The 2 CLL patients did not respond to CAR T-cell therapy (**Figure 2**).

The median (range) follow-up for the 14 study patients was 14.8 months (0.9-62.0 months). The 1-year PFS rate for all study patients was 21%; 13% for the ALL patients and 50% for the DLBCL patients. The respective 1-year OS rates for all study patients, ALL, and DLBCL were 57%, 50%, and 50%, respectively.

### Persistence of CAR T-cells in patients

**Figure 3:**
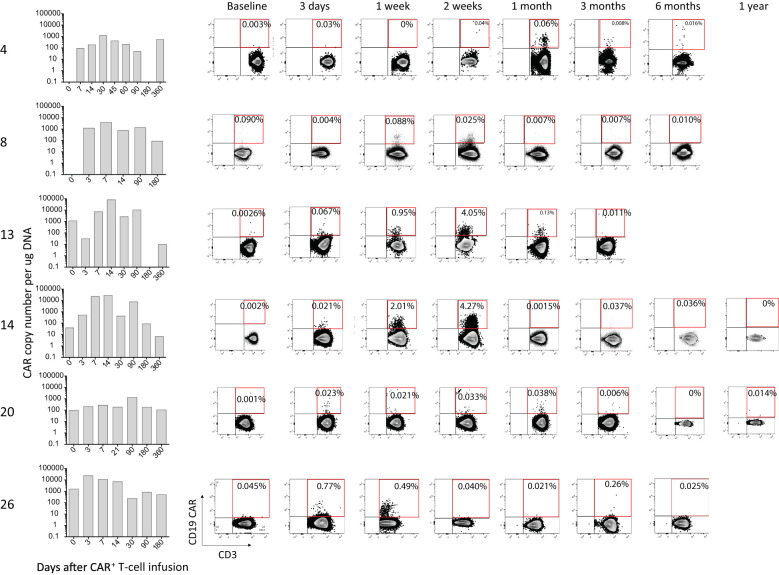
Persistence of CAR^+^ T-cells in selected patients. The presence of CAR T-cells was evaluated by ddPCR (bar graphs) and flow cytometry (dot-plots) before (baseline) and at various timepoints after infusion of the gene-modified T-cells.

Peripheral blood was collected serially over time from the patients, and the presence of genetically modified CAR^+^ T-cells was investigated using both ddPCR and flow cytometry. CAR T-cells could be detected by flow cytometry up to 30 days post infusion, after which the level of detection was at background level. Using the more sensitive ddPCR method, CAR T-cells could be detected up to an average of 203 days post infusion. Data for select patients are shown in **Figure 3**, and data for all patients is shown in **Supplementary Figure S2**. We did not observe any correlation between persistence and response.

### Serum cytokines

Persistence of CAR^+^ T-cells depends on signaling through the CAR moiety and through cytokine receptors. We observed no difference in the levels of cytokines signaling through the common cytokine receptor γ (gamma) chain before and after infusion of the T-cells. We noted low levels of IL-2 and IL-15, normal levels of IL-4 and IL-7, and elevated levels of IL-9. Of the cytokines implicated in cytokine release syndrome, IFN-γ and CXCL10 were altered 1 week (p<0.05) and 2 weeks (p<0.05) post infusion, respectively (**Supplementary Figure S3**).

## Discussion

We report on the long-term findings of non-viral, SB generated, autologous CD19 directed CAR T-cells in patients with advanced lymphoid malignancies. We note our ability to consistently manufacture up to dose level three (10^7^ CAR T-cells/kg). Furthermore, similar to our previous trial (8), the CAR T infusion was very well tolerated, with minimal rates of CRS and no noted ICANS. The intent of this trial was to investigate whether modifications to the CAR T product would increase efficacy. Our previous clinical trials infused T-cells expressing a 2^nd^ generation CAR (designated CD19RCD28) with an IgG_4_-Fc stalk that activated T-cells *via* chimeric CD28 and CD3ζ (8). Modifications to the CAR stalk to reduce binding of Fc receptor(s) and antigen recognition has shown to further improve the persistence of the genetically modified T-cells (23–25). Of the various stalks tested, including the IgG_4_-Fc mutant EQ (L235E and N297Q), CD8α hinge, and 12aa IgG_1_ hinge, the CAR with a CD8α-derived hinge (CD19RCD8CD28) showed reduced binding to Fcγ receptors (FcγR) and superior efficacy and persistence in NSG xenograft MRD leukemic models (13). Moreover, repeated stimulation cycles can erode the therapeutic potential of *ex vivo* propagated T-cells (26, 27). Therefore, we shortened the length of time in tissue culture to sustain the outgrowth of CAR^+^ T-cells that preserves a “memory” T-cell phenotype and genotype. Reducing the number of recursive stimulation cycles on aAPCs from 4x to 2x showed an improved memory phenotype (CCR7/CD45RA) (28) of the CAR T-cells, which led to improved efficacy and survival in mouse models (13).

With these modifications, we noted robust *in vivo* expansion of the CAR T product and responses in some patients. We treated a heterogeneous patient population, which precludes our ability to study any predictors for response. We noted long-term persistence in some patients but there did not appear to be any correlation between *in vivo* CAR T persistence and response.

Further modifications to the CAR T construct, as well as exploring other tumor associated antigens (TAA) and other substrates for CAR T production, are under investigation in efforts to improve the efficacy of immunotherapies using the SB platform. Magnani et al. reported on a phase I/II trial using donor-derived CD19 CAR T-cells generated with the SB transposon and differentiated into cytokine-induced killer (CIK) cells for patients with B-ALL who relapsed after allogeneic HCT (29). The product was successfully made for all 13 treated patients and was generated from a peripheral blood collection from the donor. It consisted of mainly CD3^+^ lymphocytes, with 43% CAR expression. Patients received a single dose of the CAR T product. Six of the 7 patients treated at the highest dose level had a CR or CRi, including 5 with an MRD negative response. Robust expansion was achieved in the majority of the patients. CAR T-cells were measurable by transgene copy PCR for up to 10 months. Toxicities reported included 2 patients with grade I and 1 patient with grade II CRS at the highest dose in the absence of graft-versus-host disease (GVHD), neurotoxicity, or DLT (29).

Our study highlights the success of genetic engineering of T cells based on SB nonviral gene transfer system combined with *ex vivo* expansion on AaPCs to generate CD19 CAR T cells from patients with a variety of lymphoid malignancies. By utilizing an improved CAR design and shorter *ex vivo* expansion protocol, we observed persistence of T cells by both flow cytometry and PCR in 42% of the patients, an improvement from our previous trials, with active disease. Furthermore, SB-modified CAR T cells were well tolerated and no severe CRS or ICANS were observed. Further studies to improve persistence and efficacy are warranted, and we are adapting cytokine (IL-15) co-stimulation to support T cell *in vivo* persistence and maintenance of an immature differentiation state (30).

Therefore, SB platform allows for more cost efficient and nimble construction of CAR T products. CAR DNA constructs can be easily and rapidly produced at much lower cost (10)compared to clinical grade lentivirus or retrovirus (10, 31). Outsourcing and the need for specialized handling along with limited GMP facilities for generation of viruses and long wait times due to unprecedented demand, all make the use of recombinant viruses tedious and unattractive and hence the need for alternative non-viral transduced CAR constructs. Additionally, for early proof-of-concept trials, the reduced pricing for plasmid DNA allows for speed in translating preclinical data into clinical trials. This approach may be particularly useful in the setting of immunotherapy for patients with solid tumors, where identifying optimal TAAs is critical and under active investigation. Further studies are needed to improve efficacy of these promising therapies.

## Data availability statement

The raw data supporting the conclusions of this article will be made available by the authors, without undue reservation.

## Ethics statement

The studies involving human participants were reviewed and approved by Institutional Review Board of the University of Texas MD Anderson Cancer Center. Written informed consent to participate in this study was provided by the participants’ legal guardian/next of kin.

## Author contributions

HS, SS, LC, and PK conceptualized the study. DM completed the statistical analysis. HS, SS, JM, CD, MG, MA, SO, HH, EG, DM, DP, AO, PA, JI, IK, CH, KR, RC, ES, LC, and PK enrolled patients, monitored clinical responses, completed laboratory studies, and/or analyzed data. All authors contributed to the article and approved the submitted version.

## Funding

Clinical trial was supported by Alaunos Therapeutics.

## Conflict of interest

The technology was advanced through research conducted at MD Anderson by LC. In January 2015, the technology was licensed by The University of Texas MD Anderson Cancer Center for commercial application to Alaunos Therapeutics formerly Ziopharm Oncology, Inc., and Precigen formerly Intrexon Corporation, in exchange for equity interests in each of these companies. LC and some co-authors received equity because of the licensing of this technology. From 2015 to 2021 LC was Chief Executive Officer at ZIOPHARM. The information being reported in this publication is research in which The University of Texas MD Anderson Cancer Center has an institutional financial conflict of interest. Because The University of Texas MD Anderson Cancer Center is committed to the protection of human subjects and the effective management of its financial conflicts of interest in relation to its research activities, The University of Texas MD Anderson Cancer Center has implemented an Institutional Conflict of Interest Management and Monitoring Plan to manage and monitor the conflict of interest with respect to The University of Texas MD Anderson Cancer Center’s conduct of this research. EG and LC were formerly employed by Alaunos Therapeutics and have equity ownership in the company. KR and The University of Texas MD Anderson Cancer Center have an institutional financial conflict of interest with Takeda Pharmaceutical and Affimed GmbH. KR participates on the Scientific Advisory Board for GemoAb, AvengeBio, Virogin Biotech, GSK, Bayer, Navan Technologies, and Caribou Biosciences. ES participates on Scientific Advisory Boards for Adaptimmune, Axio, Navan, Fibroblasts and Fibrobiologics, and the NY Blood Center; has licensing or patents with Takeda and Affimed; and honorarium from Bayer Healthcare Pharmaceuticals. PK has served on advisory boards for Kite and Pfizer; received research support from Amgen and Alaunos; and has been a consultant for Jazz.

The remaining authors declare that the research was conducted in the absence of any commercial or financial relationships that could be constructed as a potential conflict of interest.

## Publisher’s note

All claims expressed in this article are solely those of the authors and do not necessarily represent those of their affiliated organizations, or those of the publisher, the editors and the reviewers. Any product that may be evaluated in this article, or claim that may be made by its manufacturer, is not guaranteed or endorsed by the publisher.
