# Supplementary material for: Sleeping beauty generated CD19 CAR T-Cell therapy for advanced B-Cell hematological malignancies
Source: Front Immunol. 2022 Nov 10;13:1032397. doi: 10.3389/fimmu.2022.1032397 (PMC9684710; doi:10.3389/fimmu.2022.1032397)
Supplement: Supplementary file 1 [file DataSheet_1.docx]

**Supplement**

| **Acc#** | **Weight (kg)** | **Dose Level** | **Infusion Dose (/kg)** | **CAR^+^ Cells Infused** | **% Viability at infusion** | **Cells Manufactured** | | **Days in culture** | **% CAR** | **CAR T cells fold exp** | **% CD3** | **% CD4** | **% CD8** | **Copy number (copies/cell)** | **Cytotoxicity (NALM6, E:T=20:1)** |
| --- | --- | --- | --- | --- | --- | --- | --- | --- | --- | --- | --- | --- | --- | --- | --- |
|  |  |  |  |  |  | **Cells at Final Cryo** | **Inferred Cells Manufactured** |  |  |  |  |  |  |  |  |
| 2 | 77.2 | 1 | 1.00E+06 | 7.72E+07 | NA | 1.20E+09 | 5.35E+09 | 31 | 97.1 | 1412 | 99.59 | NA | NA | 5.256 | 62.8% |
| 4 | 94 | 1 | 1.00E+06 | 9.40E+07 | NA | 1.60E+09 | 3.20E+09 | 24 | 92.87 | 68792.59 | 98.33 | 14 | 70 | 18.2 | 57.9% |
| 6 | 72 | -1 | 7.70E+04 | 5.54E+06 | NA | 3.24E+07 | 3.24E+07 | 17 | 19.46 | 0.488 | 80.92 | NA | NA | NA | NA |
| 8 | 67.2 | 1 | 1.00E+06 | 6.72E+07 | NA | 5.40E+08 | 5.40E+08 | 21 | 76.36 | 417.77 | 85.09 | 16.4 | 48.9 | 16.6 | 25.30% |
| 9 | 35 | 2 | 2.00E+07 | 7.00E+08 | NA | 8.20E+08 | 8.20E+08 | 16 | 93 | 3000.16 | 96 | 43 | 53.6 | 35.6 | 49.20% |
| 13 | 115 | 2 | 8.70E+06 | 1.00E+09 | 100 | 1.30E+09 | 1.30E+09 | 16 | 80.24 | 124.93 | 94.99 | 29.8 | 60.1 | 9.77 | 49.70% |
| 14 | 80 | 2 | 1.00E+07 | 8.00E+08 | 100 | 1.60E+09 | 1.60E+09 | 22 | 87.02 | 61.23 | 99.8 | 11 | 81.7 | 16 | 51.40% |
| 15 | N/A | never infused | | | NA | 5.30E+09 | 5.30E+09 | 22 | 95.09 | 64.86 | 97.9 | NA | NA | Sample not good | 16% |
| 16 | 36 | 1 | 1.00E+06 | 3.60E+07 | 100 | 1.50E+08 | 1.50E+08 | 18 | 89.67 | 182.5 | 98.3 | 2.37 | 92.4 | 43.3 | 59.90% |
| 17 | N/A | never infused | | | NA | 5.10E+09 | 2.71E+10 | 27 | 81.09 | 13120.53 | 97.47 | 3.67 | 82.4 | 1.31 | 43.3% |
| 19 | 78 | 1 | 1.24E+07 | 9.67E+08 | 100 | 1.10E+09 | 1.10E+09 | 31 | 88.22 | 1246.07 | 99.59 | 0.18 | 95.9 | 1.98 | 54.70% |
| 20 | 55 | 3 | 1.47E+07 | 8.09E+08 | 99 | 1.20E+09 | 1.20E+09 | 22 | 68.49 | 12.74 | 98.28 | NA | NA | 1.47 | 50.20% |
| 21 | 91 | 3 | 1.33E+07 | 1.21E+09 | 100 | 1.28E+09 | 1.28E+09 | 23 | 90.83 | 21.019 | 99.54 | 0.12 | 96.3 | N/A | 59.39% |
| 22 | 50 | -1 | 5.70E+04 | 2.85E+06 | 96 | 5.50E+07 | 5.50E+07 | 23 | 9.34 | 0.1199 | 53.2 | 25.3 | 63.8 | N/A | N/A |
| 23 | 63 | 3 | 2.59E+07 | 1.63E+09 | 99 | 1.92E+09 | 1.92E+09 | 30 | 80.5 | N/A | 99.58 | 11.8 | 70.3 | N/A | 56.04% (10:1) |
| 24 | N/A | Never infused | | | NA | 1.10E+08 | 1.10E+08 | 34 | 7.33 | 0.048 | 86.85 | NA | NA | N/A | N/A |
| 25 | 74 | 1 | 3.79E+05 | 2.81E+07 | 100 | 9.50E+07 | 9.50E+07 | 21 | 31.53 | 3.37 | 61.51 | 1.73 | 31.1 | N/A | 30.90% |
| 26 | 112 | 1 (didnt meet dose 4 ) | 6.40E+06 | 7.17E+08 | 93.86 | 1.43E+09 | 9.02E+08 | 22 | 65.55 | 36.76 | 93.19 | 16.3 | 76.5 | N/A | 84.99% |
|  |  | N/A | 6.00E+07 | 6.72E+09 | 78.41 | 7.95E+09 | 1.04E+10 | 25 | 78.66 | 191.5 | 98.13 | 21.6 | 70.14 | N/A | 58.82% |
|  |  | Total | 6.64E+07 | 7.43E+09 | NA |  |  |  |  |  |  |  |  |  |  |

**Supplemental Table S1:** Manufacturing details of produced CAR T products, N=26. SB electroporated cells were expanded on AaPCs in presence of cytokines, cells were enumerated, phenotyped and stimulated with AaPCs every 7-10 days. CAR T cells were cryopreserved at desired dose level, and analyzed for flow cytometry, cytotoxicity (CRA) and copy number analysis (ddPCR) in in-process testing. Inferred cells depict cell number if all cells were carried forward to the next stimulation cycle (to account for in-process cryopreservation of CAR T cells). CAR T cell fold expansion, refers to CAR T cells at time of cryopreservation vs day after electroporation. For patient 26, two products were manufactured and combined for infusion. Dose Levels (DL -1: ≤ 1x10^5^/kg; DL +1 > 1x10^5^/kg but ≤ 1x 10^6^ /kg; DL +2 > 1x10^6^/kg but ≤ 1x10^7^/kg; DL +3 > 1x10^7^/kg but ≤ 1x10^8^/kg; DL +4 > 1x10^8^/kg but ≤ 1x10^9^/kg). N/A, not available.

| **Acc#** | **DaudiB2m** | | **NALM-6** | | **CD19 EL4** | | **EL-4** | | **No DNA** | **% CAR** |
| --- | --- | --- | --- | --- | --- | --- | --- | --- | --- | --- |
|  | 20:1 | 5:1 | 20:1 | 5:1 | 20:1 | 5:1 | 20:1 | 5:1 | 20:1 |  |
| 2 | 58.40 | 53.33 | 62.90 | 49.32 | 61.30 | 52.36 | 33.70 | 21.58 | 9.55 | 97.10 |
| 4 | 54.10 | 47.81 | 57.90 | 46.39 | 36.50 | 33.49 | 11.00 | 5.71 | 11.01 | 92.87 |
| 6 | N/A | N/A | N/A | N/A | N/A | N/A | N/A | N/A | N/A | 19.46 |
| 8 | 48.30 | 28.86 | 25.30 | 16.44 | 24.20 | 18.88 | 7.70 | 3.66 | 0.46 | 76.36 |
| 9**^+^** | 4.50 | 6.26 | 49.20 | 40.84 | 41.60 | 39.39 | 4.20 | 1.91 | 1.11 | 93.00 |
| 13 | 58.80 | 51.35 | 49.70 | 41.29 | 61.10 | 53.28 | 12.00 | 7.04 | -1.30 | 80.24 |
| 14 | 59.50 | 53.43 | 51.40 | 39.45 | 43.40 | 39.93 | 9.50 | 3.15 | -1.49 | 87.02 |
| 15 | 31.60 | 14.88 | 18.50 | 10.68 | 18.40 | 13.87 | 2.60 | 0.91 | -2.46 | 95.09 |
| 16 | 65.50 | 61.50 | 59.90 | 46.75 | 40.10 | 33.99 | 6.80 | 2.00 | 8.26 | 89.67 |
| 17 | 66.70 | 59.99 | 58.30 | 44.92 | 53.80 | 44.15 | 16.00 | 7.72 | 8.16 | 81.09 |
| 19 | 51.10 | 46.74 | 54.70 | 44.37 | 47.20 | 40.79 | 12.60 | 8.87 | 2.78 | 88.22 |
| 20 | 40.60 | 36.35 | 50.20 | 41.91 | 44.40 | 38.25 | 15.90 | 10.61 | 6.34 | 68.49 |
| 21 | 65.90 | 59.96 | 59.40 | 49.06 | 48.00 | 37.12 | 17.30 | 6.11 | 1.56 | 90.83 |
| 22 | N/A | N/A | N/A | N/A | N/A | N/A | N/A | N/A | N/A | 9.34 |
| 23* | 61.41 | 52.35 | 56.05 | 43.35 | 32.03 | 22.73 | 5.50 | 1.82 | -0.68 | 80.50 |
| 24 | 31.60 | 14.88 | 18.50 | 10.68 | 18.40 | 13.87 | 2.60 | 0.91 | -2.46 | 95.09 |
| 25 | 56.15 | 44.19 | 30.89 | 16.33 | 35.28 | 21.82 | 15.99 | 7.02 | 2.59 | 31.53 |
| 26_1 | 58.03 | 66.76 | 55.43 | 59.65 | 47.95 | 52.99 | 37.09 | 29.18 | N/A | 65.55 |
| 26_2 | 63.74 | 59.37 | 58.82 | 52.30 | 49.85 | 43.53 | 14.07 | 6.73 | -0.31 | 78.66 |

**Supplemental Table S2:** Cytotoxicity of patient-derived CAR T cells, at 20:1 and 5:1 E:T ratio in a standard 4h-chrommium release assay against CD19^+^ (Daudiβ2m, NALM-6, CD19^+^ EL4) and CD19^-^ (EL4) cell lines. Autologous CD19^neg^ T cells (No DNA) were also used as targets to rule out fratricide. N/A, cells not available for assay.

*, patient # 23, 10:1 and 2.5:1 (E:T) ratio is shown;

+, patient #9, Daudi cells did not perform well as the viability of tumor cells was compromised at the time of assay.

**Supplemental Figure S1:** Representative re-directed specificity of patient CD19-specific CAR T cells. Lysis of CD19^+^ tumor targets (Daudiβ2m, NALM6, CD19^+^EL4) by CAR T cells derived from Patient #14 in a standard chroimium release assay at various effector:target (E:T) ratio is shown. CD19^neg^EL-4 and CD19^neg^ autologous T cells were used as controls.

**Supplemental Figure S2:** Persistence of CAR^+^ T-cells in patients. Patients infused with genetically modified T-cells were evaluated for the presence of CAR T-cells using (left) flow cytometry and (right) ddPCR. For every time point each symbol represents an individual patient and the red lines connect the means (horizontal bars) for each time-point.

**Supplemental Figure S3:** Levels of cytokines in patient’s serum after CAR T-cell infusion. Serum isolated from peripheral blood collected post T-cell infusion was stored at -80^0^C, thawed, diluted and analyzed using Bio-Plex Pro Human Cytokine 27-plex Assay (Bio-Rad). (i) Common γ-chain receptor signaling cytokines and (ii) CRS associated cytokines. Data are shown as box and whiskers plots depicting the range of data from minimum to maximum, with horizontal lines depicting median values at each time point. Each symbol represents an individual patient at each time point. *, p<0.05 using a Student's t test.

**Supplementary Methods**

**Flow Cytometry**: Multi-color analysis was performed on CAR T-cells using anti-CD3 (Clone SK7), CD4 (Clone SK3), CD8 (Clone SK1), CD45 (Clone 2D1), CD56 (Clone B159), CD11c (Clone B-ly6), CD19 (Clone HIB19), CD14 (Clone M0P9), CD16 (Clone 3G8), CD20 (Clone L27), CD32 (Clone FL18.26), CD45RO (Clone UCHL1), CD27 (Clone L128), CD95 (Clone DX2), CD45RA (Clone HI100), CD28 (Clone CD28.2), CD62L (Clone DREG-56), CD197 (Clone G043H7), TCRαβ (Clone IP26), TCRγδ (Clone B1) and Live/Dead Fixable Aqua Dead Cell Stain in 100µL FACS Buffer for 30 minutes at 4^0^C. Expression of CD19 CAR on SB modified T cells was detected using a proprietary fluorochome conjugated anti-CD19scfv mAb [1].

**Droplet digital PCR (ddPCR):** Presence of CAR in patient derived peripheral blood after infusion was determined by ddPCR. 20 μl of multiplex PCR mixture was prepared by mixing patient PBMC gDNA with the ddPCR 2x Master Mix and TaqMan primer/probe sets, FAM labeled CAR (context sequence, 5’-ctggtcatcaccctgtactgcaaccaccggaataggagcaagcggagcagaggcggccacagcgactacatgaacatgacc-3’) and the human HEX labeled house-keeping EIF2C1 gene (5’-GAGGGCTACTACCACCCGCTGGGGGGTGGGCGCGAGGTCTGGTTCGGCTTTCACCAGTCTGTGCGCCCTGCCATGTGGAAGATGATGCTCAACATTGATGGTGAGTGGGGAGAGCTATGGAGC-3’). PCR droplets were generated, amplified and CAR copy number was calculated as described previously [2] using Digital Droplet PCR system (Bio-Rad).

1. Jena, B., et al., *Chimeric antigen receptor (CAR)-specific monoclonal antibody to detect CD19-specific T cells in clinical trials.* PLoS One, 2013. **8**(3): p. e57838.

2. Kebriaei, P., et al., *Phase I trials using Sleeping Beauty to generate CD19-specific CAR T cells.* J Clin Invest, 2016. **126**(9): p. 3363-76.
